# Fisetin induces apoptosis in uterine leiomyomas through multiple pathways

**DOI:** 10.1038/s41598-020-64871-y

**Published:** 2020-05-14

**Authors:** Jin-Woo Lee, Hyuck Jai Choi, Eun-Jin Kim, Woo Yeon Hwang, Min-Hyung Jung, Kyung Sook Kim

**Affiliations:** 1 Medical Science Research Institute, Kyung Hee University Medical Center, Seoul, 02447 Korea; 2 East-West Medical Research Institute, Kyung Hee University Medical Center, Seoul, 02447 Korea; 3 Department of Obstetrics & Gynecology, School of Medicine, Kyung Hee University, Kyung Hee University Medical Center, Seoul, 02447 Korea; 4 Department of Biomedical Engineering, College of Medicine, Kyung Hee University, Seoul, 02447 Korea

**Keywords:** Clinical pharmacology, Drug regulation

## Abstract

Although uterine leiomyomas are the most common benign uterine tumors in women, there is no effective therapy that can also preserve the uterus and maintain fertility. The work aimed to work was to discover a potential natural agent that has pharmacological activities on uterine leiomyomas with fewer adverse effects. We chose *Rhus verniciflua* Stokes (RVS) as a candidate after primary cytotoxicity testing, and analyzed the RVS components that showed pharmacological activity. Leiomyoma cells and myometrium cells were cultured from uterine tissues obtained from patients, and were treated with RVS at varying concentrations. RVS was cytotoxic in both leiomyoma and myometrium cells; however, the effects were more prominent in the leiomyoma cells. Among the bioactive components of RVS, fisetin showed significant pharmacological effects on leiomyoma cells. Fisetin showed excellent leiomyoma cell cytotoxicity and induced apoptotic cell death with cell cycle arrest. The apoptotic cell death appeared to involve not one specific pathway but multichannel pathways (intrinsic, extrinsic, MARK, and p53-mediated pathways), and autophagy. The multichannel apoptosis pathways were activated with a low concentration of fisetin (<IC_20_) and were more vigorously activated at high concentrations (>IC_50_). This is the first demonstration to show the pharmacological activities of fisetin on leiomyoma cells. These findings suggest that fisetin may be used for the prevention and treatment of uterine leiomyomas. Since fisetin can be obtained from plants, it may be a safe and effective alternative treatment for uterine leiomyomas.

## Introduction

Uterine leiomyomas are the most common benign uterine tumors in women, especially during their reproductive years^1,2^. They are monoclonal tumors of the uterine smooth muscle cells. Leiomyomas can be classified by their location relative to the layers of the uterus, such as subserous, intramural or submucous, and can be single or multiple^3,4^. Although they can occur elsewhere in the body, leiomyomas most frequently occur in the myometrium. The uterine leiomyomas do not cause pain or other symptoms in most cases. However, they very occasionally are associated with symptoms such as abnormal bleeding, pain, and pressure depending on their size and location within the uterus^5^. The exact cause of leiomyomas remains unclear. Several factors such as genetic abnormalities, alterations in growth factor expression, and abnormalities in the blood vessels have been suggested to play an important role in the development of leiomyomas^6^.

There are several uterine leiomyoma treatment options, including medications, surgery, high-intensity focused ultrasound, and uterine artery embolization^7,8^. Although the choice of treatment depends on the symptoms and leiomyoma size, surgery is currently the most common treatment. Several drugs have been used for conservative treatment, but they have been used to treat the symptoms rather than to cure the disease. Selective progesterone-receptor modulators including asoprisnil, ulipristal, and telapristone have been suggested as therapeutic drugs for uterine leiomyomas^9^. Among them, ulipristal acetate showed promising results in the treatment of leiomyomas and the control of leiomyoma-associated bleeding^10^. However, the European Medicines Agency recently reported that 5 mg daily administration has been linked with liver injury^11^. Subsequently, the Pharmacovigilance Risk Assessment Committee recommended against the prescribing of ulipristal^12^. Therefore, it is necessary to develop safer alternative methods or medications for the treatment of leiomyomas that can also preserve the uterus and maintain fertility.

In this study, we investigated a natural plant, *Rhus verniciflua* Stokes (RVS), and analyzed which of its components has a pharmacological activity for leiomyomas. RVS is an herbal medicine possessing various pharmacological effects including antioxidant, antiproliferative, anti-inflammatory, antitumor, and antimutagenic effects^13,14^. Leiomyoma cells and normal myometrium cells were cultured from tissues obtained from patients, and then treated with RVS. After confirmation that RVS was cytotoxic specifically in leiomyomas, three components of RVS including fustin, fisetin, and sulfuretin were selected as candidates for cytotoxicity studies. The apoptotic effect of fisetin on the leiomyoma cells was confirmed and the underlying mechanism of apoptosis was investigated.

## Results

### Selection of the natural product and its extracts

**Figure 1:**
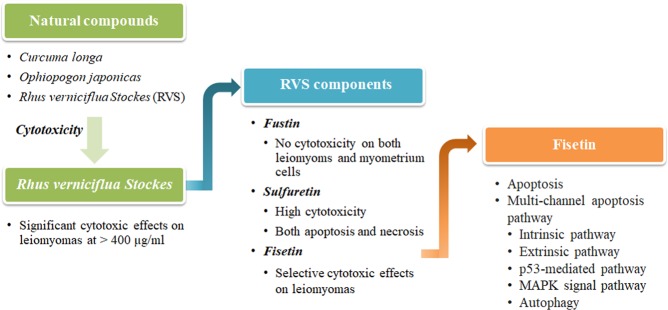
Flow chart showing the process of discovering natural products that have pharmacological effects on uterine leiomyoma cells.

The purpose of this work was to discover new agents exerting anti-uterine leiomyoma activities. A natural product that appeared to be pharmacologically active against leiomyomas was chosen, and the component showing effective pharmacological effects was confirmed. Then, the underlying mechanism of apoptosis induced by the selected component was confirmed (Fig. 1).

Firstly, three kinds of plants—*Curcuma longa*, *Orostachys japonicas*, and RVS—were selected for antileiomyoma activity assessment. *Curcuma longa*, which belongs to the family *Zingiberaceae*, is a well-known herbal medicine used in Asian countries in the treatment of many diseases such as helminthic infections, asthma, gonorrhea and urinary infections^15^. Its diverse pharmacological activities, such as anti-inflammatory, antioxidant, antibacterial, and antitumor activities, have been well documented^16–19^. *O. japonicas* also have shown various pharmacological activities, including anti-inflammatory, neuroprotective, anti-ulcerative, and anti-oxidant activities^20–22^. In particular, it has shown excellent anti-cancer effects on hepatic stellate cells, leukemia cells, colon cancer cells, and prostate cancer cells^23–25^. RVS is a well-known traditional medicinal plant that possesses a variety of pharmacological activities. It has been widely used for treating various stomach diseases and cancers^26–28^. With RVS treatment, cell growth was inhibited and apoptosis was induced in human lymphoma cells and human chronic myelogenous leukemia K562 cells^26,27^. RVS also induced apoptosis in paclitaxel-resistant ovarian cancer cells^28^.

As the first step, the cytotoxicity of the three natural plants on uterine leiomyoma cells was examined. Unexpectedly, *C. longa* showed no significant cytotoxic effects (Fig. S1A), whereas the cytotoxic effects of *O. japonicas* were prominent (Fig. S1B,C). The main components of *O. japonicas* having cytotoxic activities were quercetin, kaempferol, and epicatechin gallate, which are also among the main components of *green tea*^29^. We found several studies that demonstrated the cytotoxic effects of these components on leiomyomas^30,31^. Therefore, we focused on RVS in this work. Even though the apoptotic activities of RVS on various types of cancer cells have been well documented, no studies have investigated the pharmacological effects of RVS or of its single components such as fustin, sulfuretin, and fisetin on leiomyoma cells.

### Cytotoxic effects of RVS on leiomyoma cells and myometrium cells

**Figure 2:**
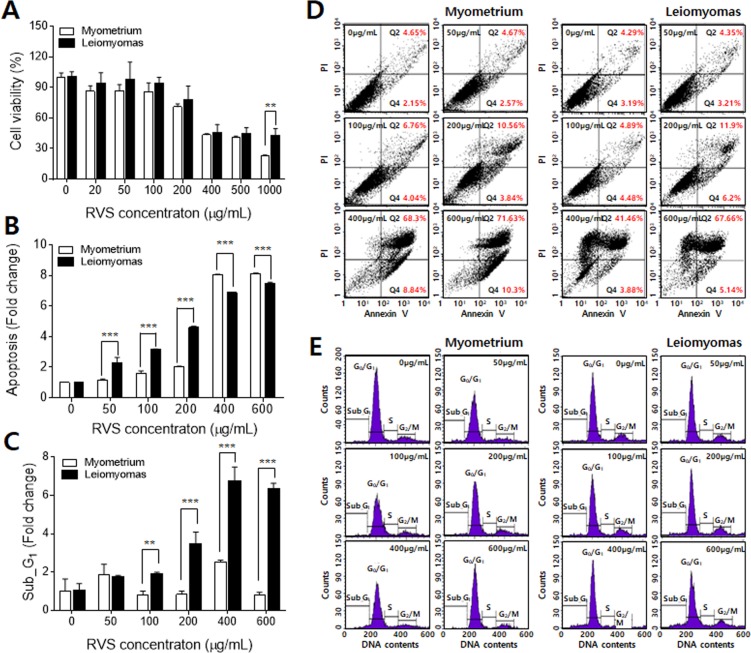
Effects of RVS on proliferation and apoptosis of leiomyoma cells and myometrium cells. (**A**) Both cells were treated with RVS at varying concentrations (0–1000 μg/mL), and cell viability was measured by the MTT assay. (**B**) The populations of cells in early and late apoptosis were counted. (**C**) Alteration of cell cycle populations (sub-G_1_) induced by RVS treatment. (**D**) Analysis of apoptosis by annexin V and PI staining in myometrium cells (left) and leiomyoma cells (right). The apoptotic status of cells is presented as dot-plots. (**E**) Cell cycle analysis in myometrium cells (left) and leiomyoma cells (right). The cells were treated with RVS (0, 20, 100, 200, 400, 600 μg/mL). Each value represents the mean ± SD (*p < 0.05, **p < 0.01, ***p < 0.001).

To evaluate the cytotoxic effect of RVS, leiomyoma cells and myometrium cells were inoculated into 60 mm culture dishes at a density of 2.5 × 10^5^ cells/dish. The cells were treated with RVS at varying concentrations (20–1,000 µg/mL), and then cultured for 24, 48, and 72 h, respectively. As shown in Fig. 2A, the viability of the leiomyoma cells decreased with increasing concentrations of RVS after 24 h treatment, and similar cytotoxic effects were observed in the groups treated for 48 and 72 h (Fig. S2A,B). The viability of the myometrium cells also decreased with RVS treatment and showed a dependency on the RVS concentrations in all groups at 24, 48, and 72 h of treatment (Figs. 2A, S2A,B). In both cell types, the cytotoxicity of RVS was significant above 400 μg/mL. In leiomyoma cells, the IC_50_ values were estimated to be 414.0 μg/mL (24 h), 278.1 μg/mL (48 h), and 249.2 μg/mL (72 h), respectively. The IC_50_ values in myometrium cells were estimated to be 370.1 μg/mL (24 h), 257 μg/mL (48 h), and 220 μg/mL (72 h), respectively.

To investigate the mechanism of cell death induced by RVS, both leiomyoma cells and myometrium cells were treated with RVS at concentrations of 50, 100, 200, 400, and 600 μg/mL and then cultured for 24, 48, and 72 h, respectively. The total percentage of apoptotic cells induced by RVS was determined with an annexin V-FITC/PI apoptosis assay. Figure 2B,D show the results of the apoptosis assay after 24 h RVS treatment in leiomyoma cells and myometrium cells, respectively. Similar proportions of cells underwent apoptosis when comparing the cell types at low concentrations (≤200 μg/mL). However, the apoptosis rate of the normal cells was remarkably increased at high concentrations: 400 and 600 μg/mL. This is an undesirable result. In cell-based experiments, treatment with higher concentrations than 400 μg/mL of a drug is considered a very harsh condition. The effect at a lower concentration, of 200 μg/mL or less, is generally considered to be clinically significant. Therefore, we think that the high sensitivity of myometrium to concentrations of RVS over 400 μg/mL is not a meaningful result. We observed a similar dependence of apoptosis on the RVS concentrations after 48- and 72-h treatment (Fig. S2C,D).

The flow cytometry analysis was performed to determine the cell cycle phase at which RVS exerts its growth inhibitory effects. The population of sub-G_1_ cells among the leiomyoma cells after 24 h of RVS treatment increased even at a low concentration of 50 μg/mL, and the rate of increase was significant at high concentrations (≥400 μg/mL) (Fig. 2C,E). In the case of myometrium cells, the population of cells in sub-G_1_ slightly increased with RVS, but the rate of increase was not significant and no concentration dependency was observed. The changes in the proportion of sub-G_1_ cells after 48 and 72 h showed a similar dependency on the RVS concentration after 24 h of treatment (Fig. S2E,F).

These results indicated that the cytotoxic activity of RVS is not only generated in leiomyoma cells but also in normal myometrium cells. Therefore, we compared the cytotoxic effects of leiomyoma cells and myometrium cells at each concentration of RVS and statistically analyzed the difference. Statistical analysis was performed using repeated measures ANOVA and Bonferroni post-test. The analysis confirmed that the cell death rate in leiomyoma cells was statistically greater than that in myometrium cells under certain conditions. Compared to myometrium cells, RVS effectively induced cell cycle arrest and inhibited the cell proliferation of leiomyoma cells at concentrations of 200 μg/mL (24 h), 50 μg/mL (48 h), and 50 μg/mL and 200 μg/mL (72 h), respectively.

### Cytotoxic effects of fisetin on leiomyoma cells and myometrium cells

**Figure 3:**
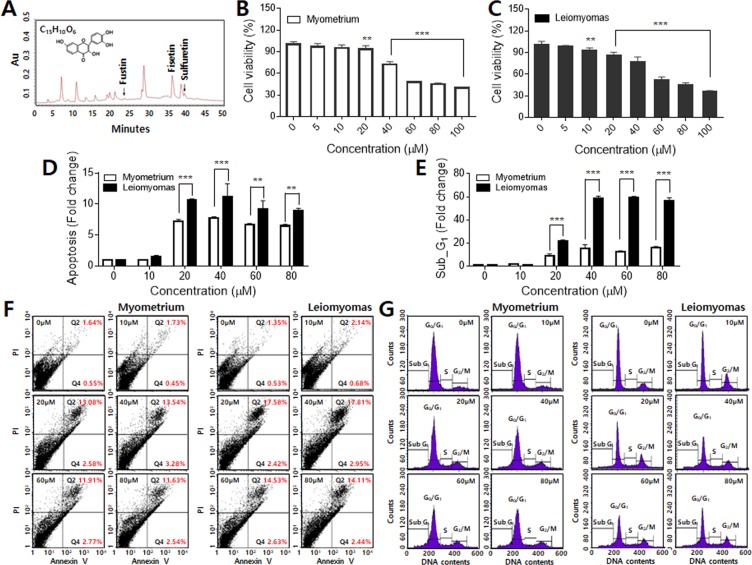
Effects of fisetin on proliferation and apoptosis of leiomyoma cells and myometrium cells. (**A**) HPLC chromatograms of RVS showed three main components: fustin, sulfuretin, and fisetin. (**B**) Myometrium cells were treated with fisetin at varying concentrations (0–100 mM), and cell viability was measured by the MTT assay. (**C**) Leiomyoma cells were also treated with fisetin, and their viability was analyzed. (**D**) The fold changes in cell numbers in both early and late apoptosis were counted. (**E**) Alteration of cell cycle populations (sub-G_1_) after fisetin treatment. (**F**) Analysis of apoptosis by annexin V and PI staining in myometrium cells (left) and leiomyoma cells (right). The apoptotic status of cells is presented as dot-plots. (**G**) Cell cycle analysis in myometrium cells (left) and leiomyoma cells (right). The cells were treated with fisetin (0, 50, 100, 200, 400, 600 mM). Each value represents mean ± SD (*p < 0.05, **p < 0.01, ***p < 0.001).

To identify causative factors of cytotoxicity in leiomyoma cells, the principal components of RVS were analyzed via HPLC. Among the chemical components of RVS, fustin, sultretin, and fisetin are the main components with anticancer and anti-inflammatory effects^29^. The RVS components we used in this study were confirmed by comparison with the HPLC results of the reference standards of fustin, sulfuretin, and fisetin. We confirmed that RVS contained 0.05% (w/w) fustin, 0.16% (w/w) sulfuretin, and 0.60% (w/w) fisetin, respectively (Fig. 3A). Since the amounts of fustin, sulfuretin, and fisetin in RVS were too small to use in our studies on viability, apoptosis, and cell cycle measurements, we purchased the three components to analyze their effects. Because the purchased fustin, sulfuretin, and fisetin each consisted of a single chemical component, there should have been no difference in composition or effect between the components directly extracted from RVS and those that were commercially obtained. Using the latter, we could exclude the possibility of involvement of foreign substances in the extracts from the laboratory.

To evaluate the cytotoxic effects of the RVS components, leiomyoma cells and myometrium cells were seeded into 96-well plates at a density of 1.0 × 10^4^ cells/well. The cells were treated with fustin, sulfuretin, and fisetin at varying concentrations and then cultured. The cytotoxic activity of fustin on myometrium cells was not significant (Fig. S3A). After 24 h of treatment, no change in cell viability was observed at concentrations up to 40 µM, and then the viability decreased by only 20% at 60 µM. The cytotoxic effect of fustin on leiomyoma cells was also insignificant (Fig. S3B). The slight decrease in cell viability depending on the concentrations was observed in all conditions after 24, 48, and 72 h treatments. Sulfuretin, however, showed more pronounced cytotoxicity. In both cell types, the cell viability decreased by more than 50% at 60 µM (Fig. S3C,D). However, the results of Annexin V-FITC/PI apoptosis assay showed that necrosis occurs as well as apoptosis by sulfuretin in leiomoyma cells. Both increased with the concentrations of sulfuretin (Fig. S4A,B). Therefore, based on the results of the MTT and apoptosis analyses, we decided not to investigate fustin and sulfuretin further.

The myometrium cells showed no changes in viability at low concentrations (≤10 µM) of fisetin. However, their viability significantly decreased at higher concentrations (≥20 µM) (Fig. 3B). The cytotoxic activities of fisetin on leiomyoma cells were very effective (Fig. 3C). The decreasing of viability was apparent from 10 µM, and the viability decreased rapidly and statistically at higher concentrations (≥20 µM).

To investigate how fisetin induced cell death, both myometrium cells and leiomyoma cells were treated at varying concentrations. The proportion of apoptotic cells increased among the myometrium cells; however, the rate of increase was lower than that of the leiomyoma cells (Fig. 3D). Among the leiomyoma cells, the population of apoptotic cells increased up to 6.4–7.7-fold after 24 h of treatment compared to the non-treated cells, respectively. However, there was no significant change in the number of necrotic cells. The difference in the proportions of apoptotic cells between the leiomyoma cells and the myometrium cells was significant at concentrations ≥ 20 µM. Figure 3F shows the detailed results of the apoptosis assays in myometrium cells (left) and leiomyoma cells (right).

In the cell cycle analysis, the proportion of myometrium cells in sub-G_1_ phase increased with fisetin treatment. However, the increase in the sub-G_1_ population was more significant in leiomyoma cells, and it was statistically significant at the fisetin concentrations of 20, 40, 60, and 80 µM. The proportion of leiomyoma cells in sub-G_1_ phase after treatment with fisetin for 24 h increased by up to 36-fold (Fig. 3E,G). These results suggest that inhibition of cell cycle progression is one of the molecular events associated with apoptotic activities of fisetin in leiomyoma cells. All measurements of cell apoptosis and cell cycle were conducted after 48 h treatment, and 24 h treatment yielded similar results (Figs. S5 and S6).

### Intrinsic & extrinsic apoptosis pathways

Generally, the cell death process can be classified into apoptosis, necrosis, autophagy, and mitotic catastrophe^30^. Among them, apoptosis, which is programmed cell death, is processed in mainly 4 different pathways: the intrinsic, extrinsic, p53-mediated, and activated MAPK pathways.

The intrinsic pathway involves mitochondrial outer membrane permeabilization (MOMP) and diverse proteins causing apoptosis induction and signal transduction^31,32^. In particular, apoptosis machinery components including the death receptor, the bcl-2 family, and caspases play major roles in the intrinsic pathway. Death receptors are cell surface receptors belonging to the tumor necrosis factor receptor gene superfamily; the death receptors trigger apoptosis upon ligand binding. The bcl-2 family of proteins, which comprises several proapoptotic multidomain proteins, initiates MOMP by forming pores at the mitochondrial outer membrane. The caspases are a family of protease enzymes that play essential roles in the initiation and progression of apoptosis. The extrinsic pathway is triggered by death receptors such as Fas and tumor necrosis factor-related apoptosis-inducing ligand receptors.

**Figure 4:**
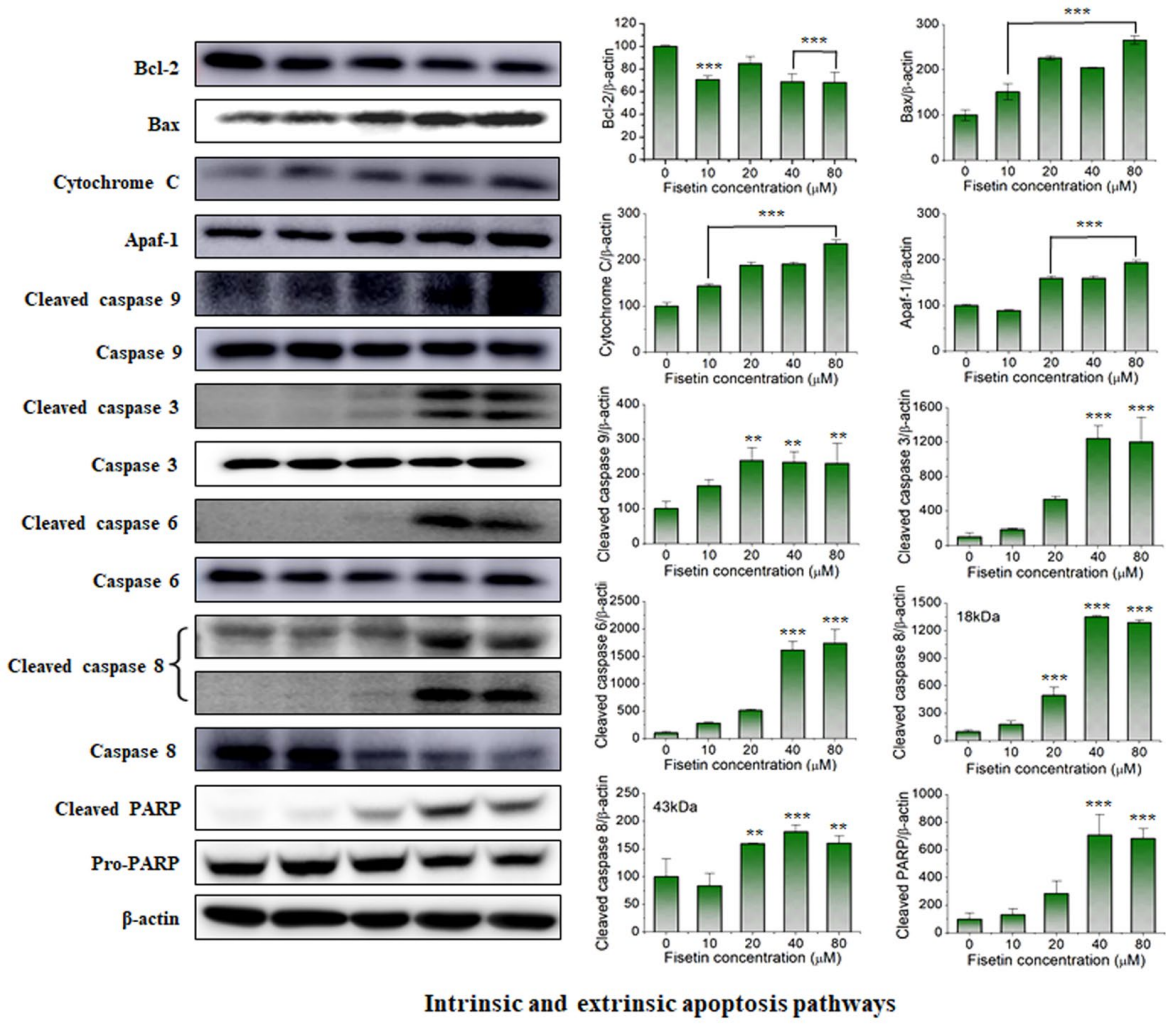
Effects of fisetin treatment on expression of Bcl-2, Bax, Cytochrome c, Apaf-1, caspase 3, 6, 8, 9 and PARP in leiomyoma cells. Leiomyoma cells were treated with fisetin for 24 h, and the expression of intrinsic and extrinsic apoptosis-regulating proteins was analyzed by western blot analysis. β-actin was used as an internal control. Results were expressed as the mean ± S.D. from three independent experiments. **p < 0.01 and ***p < 0.001 were used to indicate statistical significance compared to the untreated control cells.

Figure 4 shows the effects of fisetin on the intrinsic and extrinsic apoptosis pathways of leiomyoma cells as a function of concentration. All concentrations of fisetin except for 20 µM decreased the protein expression of bcl-2, and all decreases were statistically significant. Expression of the bax and cytochrome C proteins increased with fisetin treatment in a concentration-dependent manner. The expression levels of both apaf-1 and caspase-9 showed no change at the lowest concentration (10 µM), but significantly increased at higher concentrations (≥20 µM). Caspase-3 and caspase-6 showed increases in expression with increasing fisetin concentration, but the changes at 10 and 20 µM were not statistically significant. However, when treated at higher concentrations (30 and 40 µM), their expression levels increased rapidly. This result indicates that fisetin inhibits the activity of bcl-2 in leiomyoma cells, which results in the expression of bax and cytochrome C in mitochondria. Then, the cytochrome C is released into the cytoplasm and binds to apaf-1 and caspase-9 to form a protein complex that activates caspase-3 and caspase-6 to break down important proteins, which leads to a typical intrinsic pathway leading to death. Fisetin increased the expression of both the 18-kDa (active) and 43-kDa (intermediate) forms of caspase 8. Fisetin also increased the expression of PARP, and the increase was significant at high concentrations (≥40 µM). This result indicates that fisetin induces the death of leiomyoma cells through caspase-dependent apoptosis.

### Activation of p53-mediated apoptosis by fisetin

**Figure 5:**
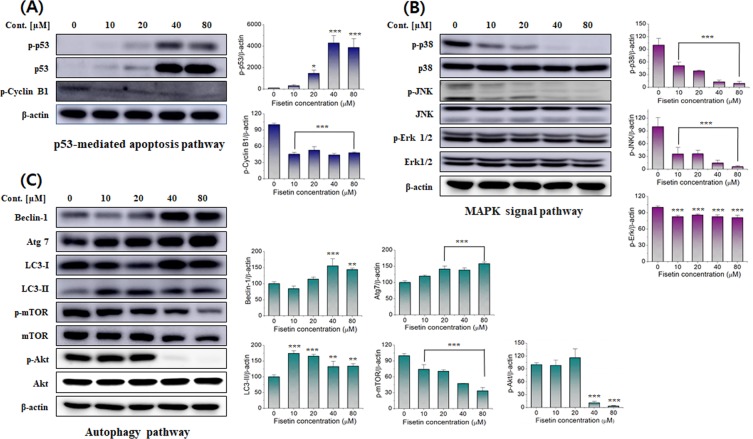
Effect of fisetin on cell cycle, MAPK phosphorylation, and autophagy flux-related protein levels in leiomyoma cells. Leiomyoma cells were treated with fisetin at 0, 10, 20, 40, and 80 μM concentrations for 24 h, and then the levels of proteins related to the apoptosis pathways were evaluated by western blot analysis. (**A**) The expression of p53 and cyclin B1 proteins changed after the fisetin treatment. (**B**) The protein levels of phosphorylated p38, JNK, and ERK were measured to understand the effects of fisetin on the pattern of MAPK phosphorylation. (**C**) To understand the effects of fisetin on autophagy, Beclin-1, Atg7, LC3 I, II, total mTOR, Akt, and the phosphorylated forms of mTOR (p-mTOR) and Akt (p-Akt) were analyzed. β-actin was used as an internal control. Results are representative of three independent experiments. *p < 0.1, **p < 0.01, and ***p < 0.001 were used to indicate statistical significance compared to the untreated control cells.

The tumor suppressor gene p53 plays a central role in the regulation of the cell cycle, apoptosis, and DNA repair^33,34^. p53 regulates the expression of various genes in response to DNA damage. The expression of p53 rapidly increases when DNA is damaged, which results in the induction of p21, one of the target genes of p53. The p21 inhibits the activity of cyclin-CDK (cyclin-dependent kinase) complexes. In normal cells, the G_2_/M transition is regulated by the cyclin B-CDK1 complex. At the end of the G_2_ phase, it is phosphorylated by the CDK-activating kinase, which leads the cell into the mitotic phase. However, p53 in damaged cells inhibits the G_2_/M transition by inhibiting the activity of the cyclin B-CDK1 complex. Fisetin increased the expression of p53 in leiomyoma cells, and the changes were evident when the fisetin concentration exceeded 20 µM (Fig. 5A). At the same time, the expression level of cyclin B decreased to less than 50% of that in untreated cells. These results are consistent with the changes in the proportions of the leiomyoma cells in the sub-G_1_ (Fig. 3E) and G_2_/M phases (Fig. S6D,E). Therefore, it is thought that apoptosis of leiomyoma cells is also caused by p53-induced cell cycle arrest.

### Activation of the MAPK pathway by fisetin

The mitogen-activated protein kinase (MAPK) family members form a crucial signaling pathway for the maintenance of cells against external stress^35,36^. The MAPK family consists of three main subfamilies: the extracellular signal-regulated protein kinases (ERKs), the c-Jun N-terminal kinases (JNKs), and the p38-MAPKs. JNKs and p38-MAPKs are activated by proinflammatory cytokines, UV irradiation, heat, osmotic shock, hydrogen peroxide, and DNA damage, and help to regulate growth inhibition or apoptosis induction. ERKs are activated by mitogenic stimuli such as growth factors, cytokines, and phorbol esters, and help to regulate cell growth and differentiation.

With fisetin, there were no changes in total ERK, JNK, and p38-MAPK protein expression (Fig. 5B). However, the expression levels of their phosphorylated forms showed definite changes induced by fisetin. The expression levels of phospho-p38 and phospho-JNK decreased significantly in a concentration-dependent manner, but the decrease of phospho-ERK showed no concentration dependency. These results suggest that the MAPK pathway was activated by fisetin and played a crucial role in the apoptosis of leiomyoma cells.

### Activation of autophagy signaling pathway by fisetin

Autophagy is a self-degradative process by which dysfunctional cellular components are degraded inside the cell and delivered to the lysosome^37–39^. Autophagy has several stages, including induction, vesicle nucleation, vesicle elongation, retrieval, docking/fusion, and vesicle breakdown/degradation. Autophagy can be initiated by inhibition of mTOR activity, during which phosphorylation of Atg13 is suppressed and a complex with Atg1 and Atg17 is formed. Class III PI3K (phosphatidylinositol 3-kinase) plays an important role in the early stages of vesicle nucleation. Its activity is determined by the formation of a multiprotein complex of Beclin-1 (Atg6), UV irradiation resistance-associated tumor suppressor gene (UVRAG), and myristylated kinase (Vps15 or p150). The autophagosome formation is regulated by Atg proteins, such as the Atg12-Atg5 and LC3-II (Atg8-II) complexes. Atg12 is conjugated to Atg5 in a ubiquitin-like reaction, which requires Atg7 and Atg10 (E1- and E2-like enzymes, respectively), in the vesicle elongation stage. The Atg12-Atg5 conjugate interacts non-covalently with Atg16 to form a large complex. LC3-I is generated by cleaved LC3/Atg8 and conjugated to phosphatidylethanolamine (PE) by Atg7 and Atg3 (E2-like enzymes) in a ubiquitin-like reaction. The binding of PE results in the formation of LC3-II (autophagic vesicle-associated form), which is a lipidated form of LC3. LC3-II is considered a marker of autophagosome formation.

**Figure 6:**
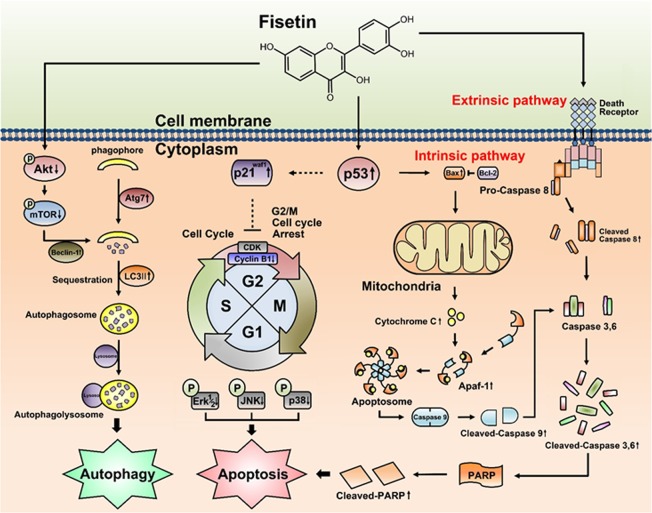
Schematic diagram of the proposed molecular mechanisms of fisetin-induced G2/M arrest, apoptosis, and autophagy in uterine leiomyomas. The schematic diagram delineates the extrinsic (death receptor) and intrinsic (mitochondrial) pathways of fisetin-induced apoptosis. Fisetin may induce apoptosis through both pathways. Mitochondria act as major control points involving the regulation of apoptosis. Uterine leiomyomas cells were exposed to fisetin, and the phagosomes were converted to double-layered membranes of autophagosomes through increasing expression levels of Atg proteins including Beclin-1and Atg-7, while LC3-I is converted to LC3-II. The inhibition of the Akt/mTOR signaling pathway contributes to the accumulation of LC3-II, which suggests that the pathway is upstream of fisetin-induced autophagy. Once the autophagosome develops, its maturation is complete upon fusion with a lysosome to form an autophagolysosome. Eventually, fisetin induces programmed cell death. In addition, phosphorylation of p53 stimulates its activation of p21, resulting in cell cycle arrest in G2/M through inhibiting cyclin B1 (CCNB1) expression and activity.

Figure 5(C) shows the changes in autophagy-related signals induced by fisetin. In the case of Beclin-1, the change with the lowest concentration of fisetin was not clear, but at concentrations of 20 μM or more, the expression level of Beclin-1 was significantly higher compared to the control group. The expression of Atg7 was increased in a concentration-dependent manner, while phospho-mTOR was decreased. The LC3-II expression level sharply increased with 10 μM fisetin and slightly decreased with a higher concentration of fisetin, but the expression level of LC3-II in all treated groups was higher than in the control group. In the case of phospho-Akt, the expression level slightly increased when the concentration of fisetin was 20 µM, but decreased sharply over 40 µM. This result suggests that autophagy occurred in the leiomyoma cells treated with fisetin. The multi-channel apoptosis pathways induced by fisetin are delineated in a schematic diagram in Fig. 6.

## Discussion

There have been few studies on the treatment of uterine leiomyomas, other than surgical treatment, over the past 100 years. Because it is a benign tumor, unlike cancer, no widely recognized cell lines and no animal study models have been developed. Therefore, it is very difficult to carry out preliminary studies including *ex vivo* experiments, which are necessary before human studies can be performed. However, when natural products are employed, there is an advantage in that human subjects can be relatively easily tested under safe conditions. In this study, we have identified a natural component showing therapeutic effects specifically in leiomyoma cells compared with normal myometrium cells. Both leiomyoma cells and myometrium cells were cultured from uterine tissues obtained from patients.

To discover agents in natural plants that have pharmacological activities targeting leiomyomas, we screened *Curcuma longa*, *O. japonicas*, and RVS, which are well-known herbal medicines in Asian countries, using an MTT assay. Based on the results, we chose RVS as a candidate for further study. RVS is a tree that belongs to the *Anacardiaceae* family, also commonly known as the *lacquer* tree. RVS has been used as a folk herbal medicine in Asian countries for a long time, and its various pharmacological activities have been revealed in recent studies^13,14^. RVS possesses several bioactive compounds including fustin, fisetin, gallic acid, butein, butin, sulfuretin, quercetin, coumaric acid, kaempferol-3-O-glucoside, and kaempferol, which are mediators of the pharmacological activities of RVS^40^.

Among them, fisetin (3,7,3,4-tetrahydroxyflavone) is a naturally occurring flavonoid found not only in RVS but also in various fruits and vegetables such as strawberries, apples, and persimmons.^41^. Fisetin has been reported to induce apoptosis in cells from various cancers such as human non-small cell lung cancer, liver cancer, prostate cancer, and laryngeal cancer, all through apoptosis signaling pathways^42^. In addition, fisetin can attenuate isoproterenol-induced cardiac ischemic injury, activate anti-inflammatory activity by inhibition of c-Jun N-terminal kinase and nuclear factor κB pathways, and induce the expression of heme oxygenase-1, which is a major component of cellular antioxidant enzymes^43^. However, to date, no study has been conducted to identify the pharmacological activities of fisetin in uterine leiomyoma cells.

We firstly demonstrated that fisetin was cytotoxic for uterine leiomyoma cells. It induced apoptotic cell death and cell cycle arrest. The fisetin-induced apoptosis was not mediated specifically by a single pathway, but by all known apoptosis pathways including intrinsic, extrinsic, MARK, p53-mediated pathways, and autophagy. This is in strong contrast with apoptosis caused by a specific drug, which usually occurs along one or two pathways. The multichannel apoptosis pathways were activated even at low concentrations of fisetin. Most of the expression levels of proteins associated with intrinsic and extrinsic pathways, including Bax, Bcl-2, caspase 8 and 9, Apaf-1, and cytochrome C, increased with fisetin treatment at the concentration of 20 µM, which is even lower than the IC_20_ (26.0 µM). The activation of p53 and deactivation of cyclin B were remarkable with 20 µM fisetin. The proteins associated with p53-mediated apoptosis and the MRAR pathway also increased with 20 µM fisetin. The expression of proteins associated with autophagy, except Akt, was also significant at 20 µM. As expected, the activation or deactivation of proteins related to apoptosis was more prominent at higher concentrations, 40 and 60 µM. Note that the IC_50_ of fisetin for leiomyoma cells is 64.52 µM.

Uterine leiomyoma is the most common tumor in women. It is found in approximately 25%–35% of women of childbearing age and 40–50% of women over 35 years old. The cause of uterine leiomyomas is not yet known exactly, but it is thought that one of the cells forming the uterine smooth muscle abnormally proliferates to form the leiomyoma. A leiomyoma is a type of hormone-dependent tumor, which is especially affected by follicular hormones and estrogen. Previously identified molecular biologic abnormalities of uterine leiomyoma include increased estrogen and progesterone receptors, bcl-2, and aromatase cytochrome P450^44^. The overexpression of transforming growth factor beta, heparin-binding growth factor, insulin-like growth factor, and basic fibroblast growth factor has been reported to play a key role in the development of uterine leiomyomas^45^. A gonadotropin-releasing hormone agonist is widely used as typical drug therapy for uterine leiomyomas, but its effect is temporary and there may be adverse effects due to estrogen deficiency^46^. Other potential medications include anti-progesterone agents, interferon, estrogen receptor modulators, and anti-fibrosis agents, but they are still in the research stage. Fisetin can be considered as a potential therapeutic agent for uterine leiomyomas because it showed cytotoxicity through multi-channel apoptosis pathways. Also, a lack of significant side effects is expected because it is being used as a dietary supplement.

In conclusion, we investigated which natural products showed pharmacological effects on uterine leiomyoma cells, and chose to study RVS after cytotoxicity analysis. Among the bioactive components of RVS, fisetin showed significant cytotoxicity in leiomyoma cells. Leiomyoma cells treated with fisetin underwent apoptotic cell death through a multi-channel pathway. This study is the first to demonstrate the pharmacological activity of fisetin on uterine leiomyoma cells.

## Materials and methods

### RVS extracts and treatment conditions

RVS was purchased from Kyung Hee Herb Pharm (Wonju, Gangwon Province, South Korea). A sample of 700 g of RVS was precisely weighed and heated at 180 °C for 1 h, 10 times the volume of distilled water was added, and then the mixture was extracted at 100 °C for 2 h. The obtained extracts were filtered under reduced pressure (EYELA A-1000S, EYELA, NY, USA) and concentrated using a rotary vacuum concentrator (BUCHI Rotavapor R-220, BUCHI Labortechnik, Switzerland) at 60 °C for 1 h. The resulting concentrates were lyophilized using a lyophilizer (Ilshin Biobase, Gyeonggi-do, South Korea) for 3 days at −80 °C, and 24 g of dried RVS was obtained. For the experiment, the dried RVS was added to the distilled water to yield a concentration of 1 mg/mL and dissolved in a mixing agitator for 4 h. The supernatant was transferred and the pellet was centrifuged at 12,000 rpm using a microcentrifuge three times, and then it was filtered using a 0.22-μM filter.

### High-performance liquid chromatography (HPLC)

HPLC analysis was performed with an Alliance 2690 Separations Module with a Waters 996 Photodiode Array Detector and Millennium 32 Chromatography Manager Version 3.2 (Waters, MA, USA). For preparative HPLC, a Nucleosil C18 column was used (5 μm, 4.0 mm × 250 mm I.D.; Macherey-Nagel, Germany). Acetonitrile, methanol, and water (J.T. Baker, USA) were used for separation. For simultaneous analysis of fustin, fisetin, and sulfuretin, the mobile phase consisted of 2% acetic acid (A) and methanol (B). The flow rate was fixed at 1.0 ml/min and the wavelength was set at 254 nm. The gradient elution was as follows: 5% B for 0 min, 20% B for 10 min, 60% B for 40 min, and 80% B for 50 min. For analysis of the sample, 10 ml of methanol was added to 100 mg of the extract, and the resulting mixture was sonicated for 30 min and then filtered through a 0.45 μm membrane filter. Fisetin used as a reference standard was purchased from Sigma (MO, USA), and fustin and sulfuretin were purchased from the company (Indofine Chemical Co Inc, NJ, USA).

### Isolation and expansion of leiomyoma cells and normal myometrium cells

Uterine leiomyomas and normal myometrium tissues were obtained from patients who underwent a hysterectomy, under approval of the institutional review board of Kyung Hee University Hospital (IRB No. KUH 2017-11-064). Written informed consent was obtained from all participants before using their tissues. All experimental methods were performed following the guidelines of the IRB. Tissue was immersed in phosphate-buffered saline (PBS, pH 7.4) supplemented with 1% antibiotics/antimycotic (Gibco BRL, Grand Island, NY, USA). The tissue was cut into 1–2 mm pieces, and enzymatically digested for 3–4 h at 37 °C in sterile Hanks’ Balanced Salt Solution (HBSS) supplemented with 2 mg/mL collagenase type I (Sigma-Aldrich, St. Louis, MO, USA) and 0.2 mg/mL DNase (Sigma-Aldrich, St. Louis, MO, USA). Next, 20% fetal bovine serum (FBS) was added to the fully digested tissue to stop the enzyme action. The tissue digest was then filtered through a 70 µm cell strainer (SPL, Gyeonggi-do KOREA) and centrifuged at 2,500 rpm for 15 min. The pellets were collected, washed with PBS, and centrifuged again. The fully filtered samples were incubated in Dulbecco’s modified Eagle medium/nutrient mixture F-12 media (Pan Biotech, Aidenbach, Germany) supplemented with 10% fetal bovine serum for 48 h at 37 °C and 5% CO_2_. The media was changed every 2 days. Cultured cells were immunohistochemically stained to visualize smooth muscle characteristics, and 99% or more pure muscle cells were identified. Only the first to third passages of cells were used in the experiment.

### MTT assay

Both leiomyoma cells and myometrium cells were incubated on 96-well plates (1 × 10^4^ cells/well) for 24 h. After the incubation, the cells were treated with RVS, fustin, fisetin and sulfuretin, separately. The concentrations of RVS were 0, 50, 100, 200, 400, 500, and 1000 µg/mL. The concentrations of fustin, fisetin and sulfuretin were 0, 10, 20, 40, 60, 80, and 100 µM. Cell viability was analyzed using a methylthiazol tetrazolium (MTT) assay after 24, 48, and 72 h of treatment. Briefly, an MTT solution (2 mg/mL) was added to each well, and the cells were incubated at 37 °C and 5% CO_2_ for 2 h. After the incubation, the MTT was aspirated and 200 μl per well of DMSO was added to each well, and then the plates were shaken for 5 min. Subsequently, the cell viability was assessed by measuring the absorbance at 570 nm using a spectrophotometric microplate reader (Molecular Devices, Sunnyvale, CA, USA).

### Annexin V-FITC/PI apoptosis assay

Cells were seeded onto 60-mm culture dishes at a density of 2.5 × 10^5^ cells/dish and incubated for 48 h at 37 °C and 5% CO_2_. After the incubation, the cells were harvested with trypsin-EDTA solution (JBI, Seoul, Korea), immersed in 5% FBS to stop the enzyme reaction, and then washed twice with PBS buffer. The apoptotic effects of RVS, fustin, fisetin, and sulfuretin were analyzed by using an annexin V-fluorescein isothiocyanate (FITC)/PI apoptosis detection kit (BD Pharmingen, San Diego, CA, USA), which contains a binding buffer, annexin V-FITC, and PI staining buffer. Briefly, leiomyoma cells and myometrium cells were suspended in 100 μl binding buffer and sequentially mixed with 5 μl annexin V-FITC and 5 μl PI. The mixture was incubated for 15 min at room temperature in the dark. Cell apoptosis was quantitated with flow cytometry (FACSCalibur, Becton Dickinson, Franklin Lakes, NJ, USA) and CellQuest 6.0 software (Becton Dickinson, Franklin Lakes, NJ, USA).

### Cell cycle analysis

For analysis of the sub-G_1_ DNA content, cells were collected by trypsinization and fixed with ethanol (70%) overnight at 20 °C. The cells were resuspended in PBS buffer containing 10 μg/ml RNase and incubated for 30 min at 37 °C. PI at 50 μg/ml was used to stain cells in the dark at room temperature for 30 min. The cell cycle phase was identified based on DNA content using a FACSCalibur flow cytometer (Becton Dickinson, New Jersey, USA) and CellQuest 6.0 software.

### Western blot analysis

Uterine leiomyoma cells were seeded in 6-well plates and incubated with fisetin at varying concentrations. The cells were collected and lysed with ice-cold lysis buffer (20 mM Tris-HCl pH 7.5, 2.5 mM sodium pyrophosphate, 1 mM EGTA, 150 mM NaCl, 1 mM Na_2_ EDTA, 1 mM β-glycerophosphate, 1 mM Na_3_VO_4_, 1 mM phenylmethylsulfonyl fluoride, 1 μg/mL leupeptin, 1% Triton) and placed on ice for 5 min. Subsequently, the lysates were centrifuged for 10 min at 12,000 rpm. The supernatants were collected and the protein concentrations in the cell lysates were determined using BCA assay kit (Thermo Fisher Scientific, Waltham, MA, USA). Protein (20 μg) was mixed with loading buffer, boiled for 5 min, and loaded onto 8%–15% polyacrylamide gels. Electrophoresis was then carried out, and the proteins were transferred to polyvinylidene difluoride membranes. The membranes were blocked with skimmed milk at room temperature for 1 h, and incubated with the antibodies at 4 °C overnight. The antibodies were listed in Table 1. Membranes were subsequently incubated with secondary antibody at room temperature for 1 h. After a second wash with TBS-T, target protein bands were visualized using an Enhanced Chemiluminescence kit (Thermo Scientific, Rockford, IL, USA). The amount of protein expression was quantified with an Amersham Imager 600 chemiluminescence imaging system (Davinch-K, Seoul, Korea).

**Table 1:** List of the antibodies, catalog number, and dilution used in the analysis of apoptosis pathways.

	Antibody	Catalog number	Dilution
Primary antibodies	anti-Akt	#4085	1:1,000
	anti-phospho Akt	#4060	1:1,000
	anti-Apaf-1	#8723	1:1,000
	anti-Bax	#2772	1:1,000
	anti-Bcl2	#2876	1:1,000
	anti-caspase 3	#9665	1:1,000
	anti-cleaved caspase 3	#9661	1:1,000
	anti-caspase 6	#9762	1:1,000
	anti-cleaved caspase 6	#97611	1:1,000
	anti-caspase 8, anti-cleaved caspase 8	#9746	1:1,000
	anti-caspase 9	#9508	1:1,000
	anti-cleaved caspase 9	#52873	1:1,000
	anti-PARP	#9542	1:1,000
	anti-cleaved PARP	#5625	1:1,000
	anti-Erk1/2	#4695	1:1,000
	anti-phospho Erk1/2	#4370	1:1,000
	anti-p38	#8690	1:1,000
	anti-phospho p38	#4511	1:1,000
	anti-JNK	#9665	1:1,000
	anti-phospho JNK	#9661	1:1,000
	anti-phospho cyclin B1	#4133	1:1,000
	anti-cytochrome c	#4272	1:1,000
	anti-p53	#9282	1:1,000
	anti-phospho p53	#9284	1:1,000
	anti-Beclin-1	#3495	1:1,000
	anti-Apaf-1	#8723	1:1,000
	anti-mTOR	#2972	1:1,000
	anti-Atg7	#8558	1:1,000
	anti-LC3A/B I and II	#12741	1:1,000
	anti-rabbit β actin	#4970	1:1,000
Secondary antibody	horseradish peroxidase-conjugated goat anti-rabbit immunoglobulin G	#7074	1:2,500

All antibodies were purchased by Cell Signaling Technology (Danvers, CO, USA).

### Statistics

All data are expressed as means ± standard deviations and were statistically analyzed by GraphPad Prism (version 5.01; GraphPad Software, San Diego, CA). The statistical significance of the difference between the control and experimental groups was determined by one-way Analysis of Variance. P-values less than 0.01 were regarded as statistically significant.

## Supplementary information

Supplementary information
