# Supplementary material for: Fisetin induces apoptosis in uterine leiomyomas through multiple pathways
Source: Sci Rep. 2020 May 14;10:7993. doi: 10.1038/s41598-020-64871-y (PMC7224361; doi:10.1038/s41598-020-64871-y)

Fisetin induces apoptosis in uterine leiomyomas through multiple pathways

Jin-Woo Lee[1], Hyuck Jai Choi[2], Eun-Jin Kim[2], Woo Yeon Hwang[3], Min-Hyung Jung[3†], Kyung Sook Kim[4†]

[1]Medical Science Research Institute, Kyung Hee University Medical Center, Seoul 02447, Korea

[2]East-West Medical Research Institute, Kyung Hee University Medical Center, Seoul 02447, Korea

[3]Department of Obstetrics & Gynecology, School of Medicine, Kyung Hee University, Kyung Hee University Medical Center, Seoul 02447, Korea

[4]Department of Biomedical Engineering, College of Medicine, Kyung Hee University, Seoul 02447, Korea

Supporting materials

Fig. S1 (A) Cytotoxic effects of *Curcuma longa* on leiomyoma cells. The leiomyoma cells were treated with *Curcuma longa* at varying concentrations (0, 20, 50, 100, 200, 400. 500, 1000 µg/mL) for 24, 48, and 72 h, respectively. It had no significant cytotoxic effects in any of the treatment conditions. Therefore, *C. longa* was not used in the viability analysis of myometrium. (B, C) The cytotoxicity of *O. japonicas* was analyzed in both myometrium and leiomyoma cells. The cells were treated with *O. japonicas* at varying concentrations (0, 20, 50, 100, 200, 400. 500, 1000 µg/mL) for 24 and 48 hours, respectively. The *O. japonicas* showed significant cytotoxicity at high concentrations (> 100 µg/mL) in both cell types.

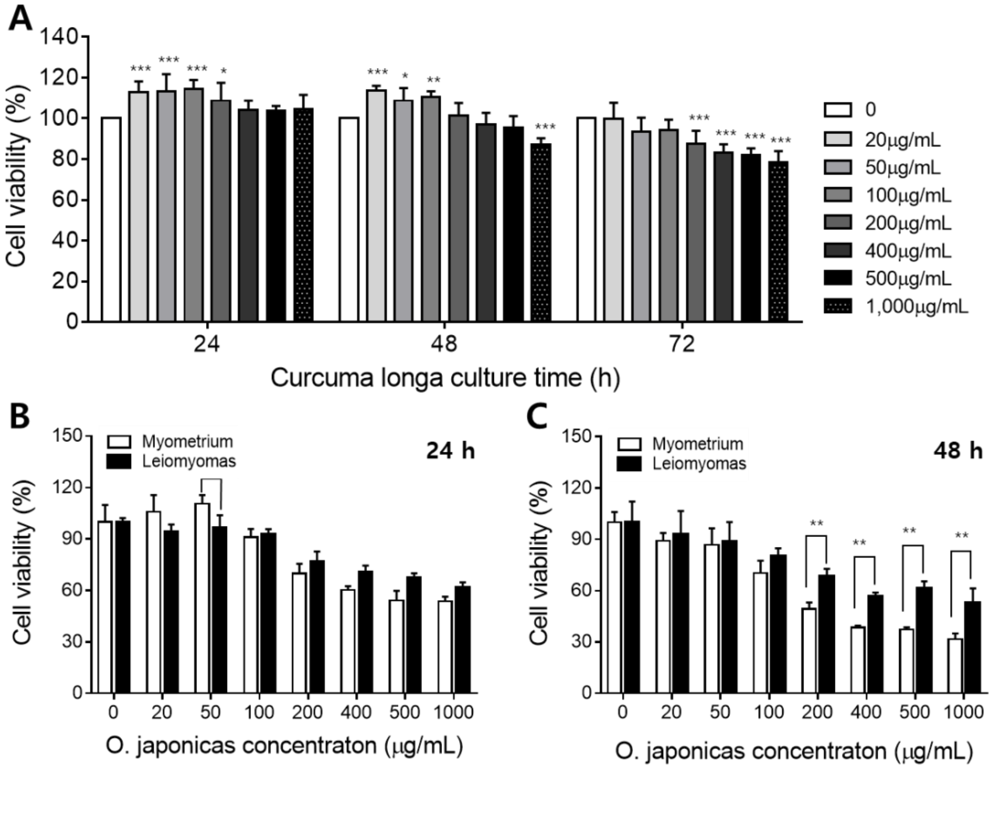

Fig. S2 RVS-induced changes in proliferation and apoptosis of leiomyoma cells and myometrium cells. (A, B) Both types of cells were treated with RVS for 48 and 72 h at varying concentrations (0–1000 μg/mL), and then cell viability was measured by the MTT assay. (C, D) The changes in the number of cells in early and late apoptosis were counted after 48 and 72 h RVS treatment. (E, F) The population of cells in the sub-G_1 stage was increased in a concentration-dependent manner in both cell types after 48- and 72-h treatments. Compared to the myometrium cells, the proportion of leiomyoma cells in sub-G_1 was larger at all RVS concentrations.

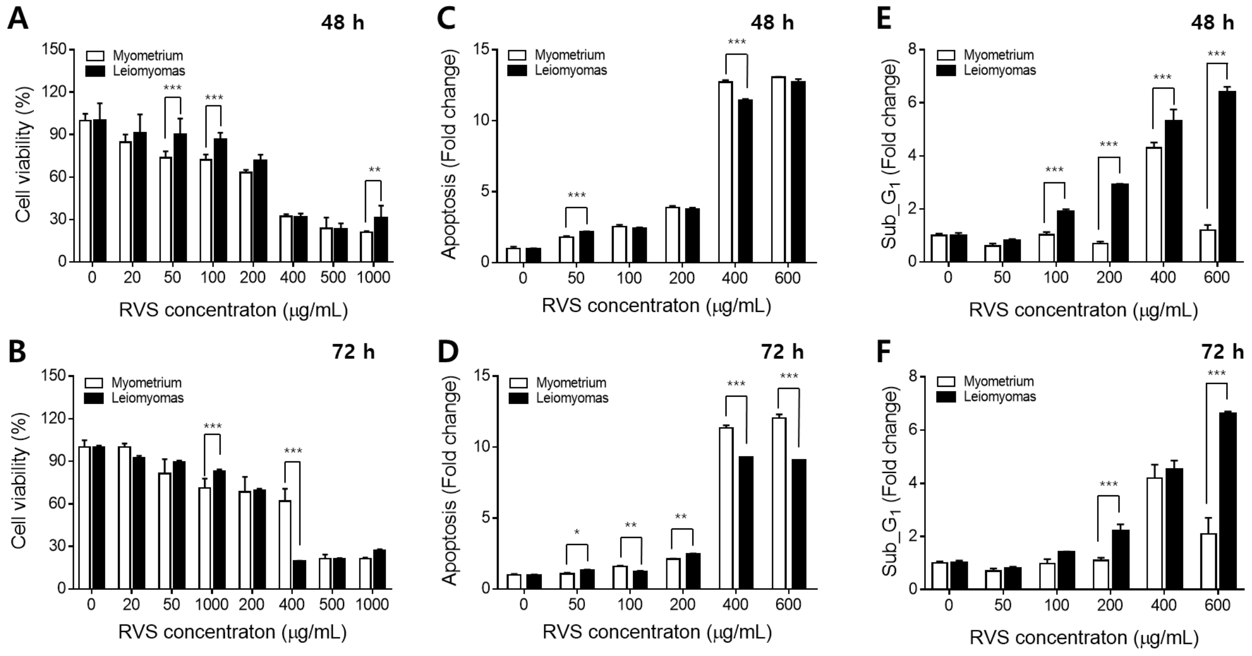

Fig. S3. Cytotoxic effects of fustin and sulfuretin on leiomyoma cells and myometrium cells. To identify cytotoxicity associated with fustin and sulfuretin, the cells were treated with each agent at varying concentrations (5, 10, 20, 60, 80, and 100 μM). (A, B) Fustin showed no significant cytotoxic effects on cells of either type in all conditions after 24-, 48-, and 72-h treatments. (C, D) Sulfuretin showed a cytotoxic effect on both cell types, and the effects were significant at concentrations over 40 μM.

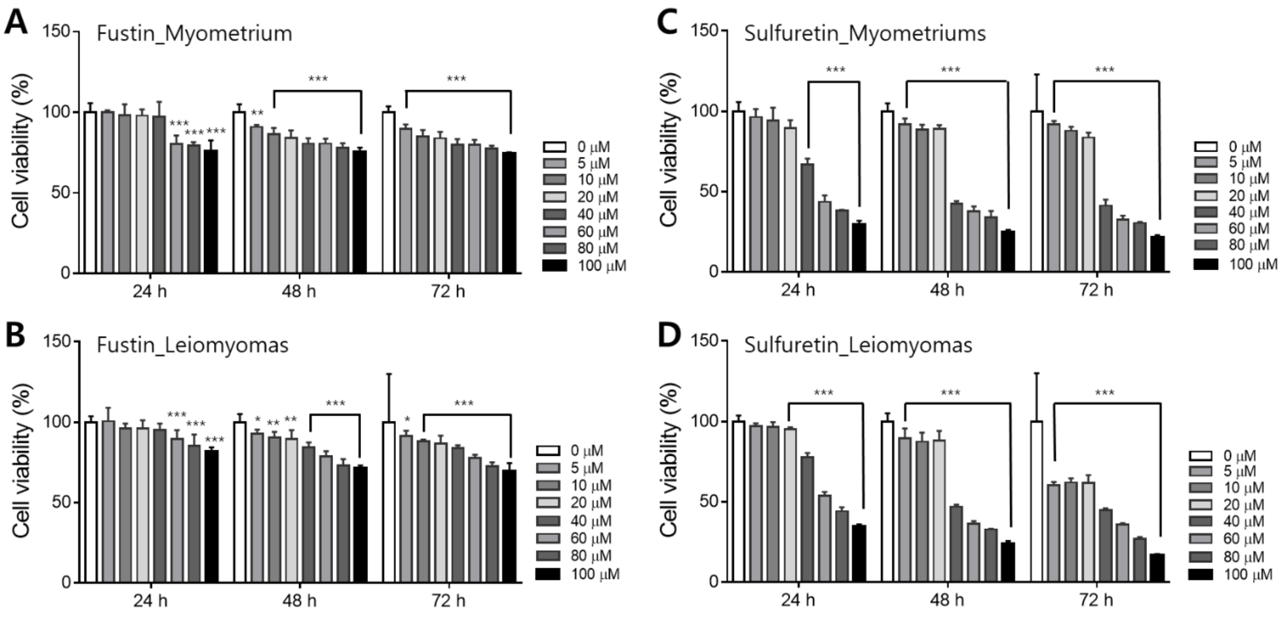

Fig. S4 (A) Effects of sulfuretin on apoptosis of leiomyoma cells and myometrium cells. The populations of apoptotic cells were increased in a concentration-dependent manner in both cell types. The difference between the apoptotic populations of leiomyoma cells and myometrium cells was significant at the concentration of 80 μM. (B) The population of necrotic cells increased rapidly with the concentration of sulfuretin.

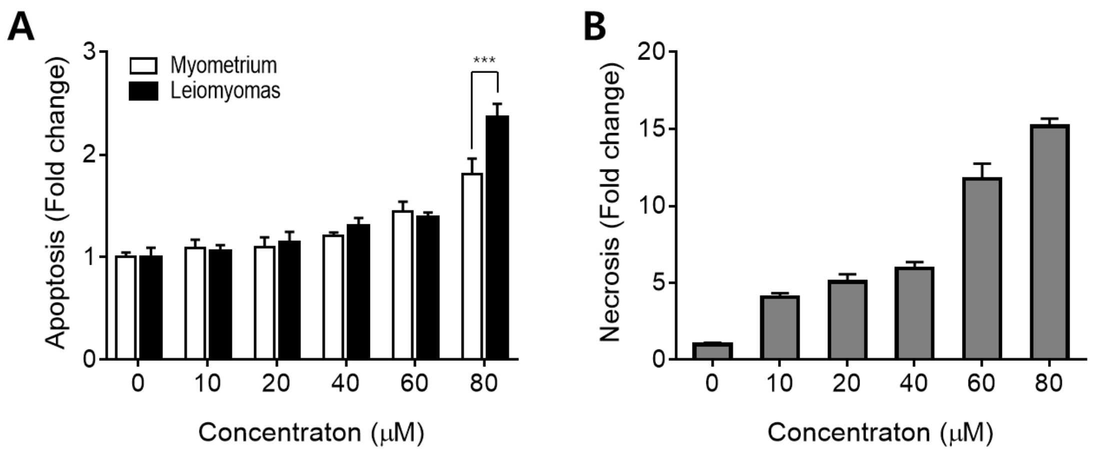

Fig. S5 Cytotoxic effects of fisetin on leiomyoma cells and myometrium cells. The change in cell viability was analyzed after 48 and 72 h. In both (A) myometrium and (B) leiomyoma cells, the viability decreased with increasing fisetin concentrations.

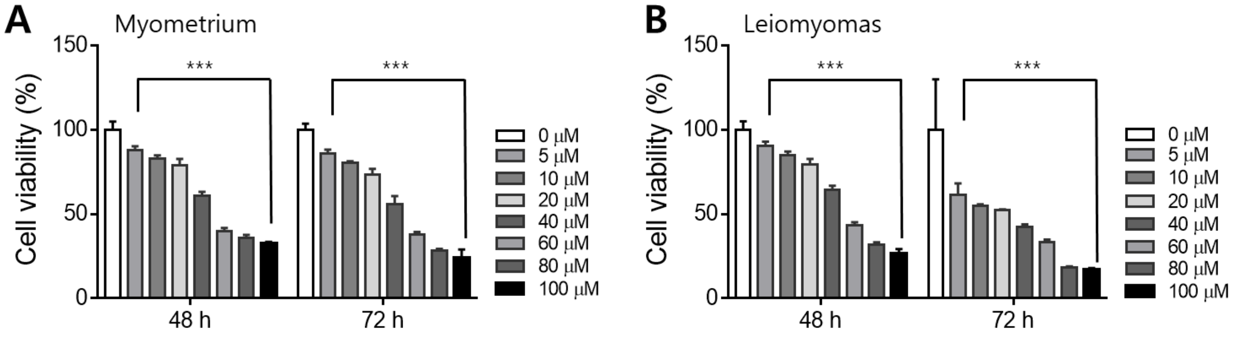

Fig. S6 (A) Changes in the population of apoptotic cells after 48-h fisetin treatment. The proportion sharply increased with 20 μM fisetin in both leiomyoma cells and myometrium cells. The difference in the proportions between the leiomyoma cells and the myometrium cells was statistically significant at the concentrations of 20 and 40 μM. Change in cell cycle induced by fisetin was analyzed after 24 h in myometrium cells and leiomyoma cells. The effect of fisetin on the cell cycle was analyzed after (B,C) 24- and (D,E) 48-h treatment. The changes in the cell cycle were similar after 24- and 48-h treatment in the two types of cells.

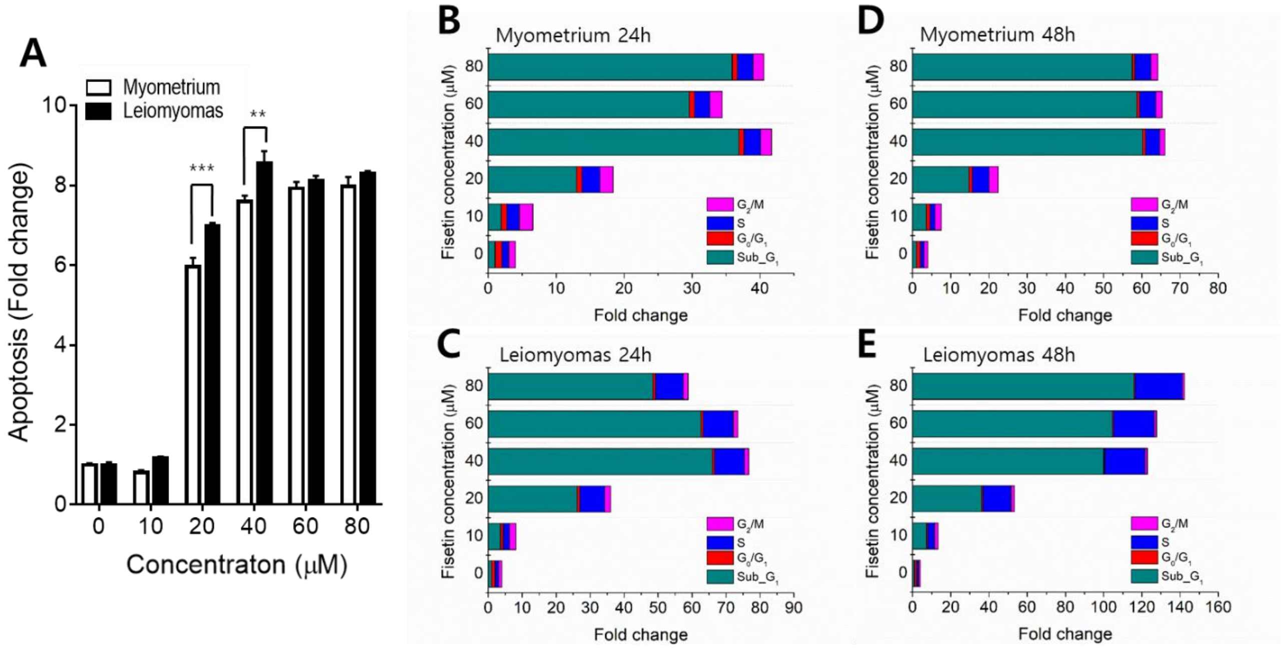

Fig. S7 Full-length blots corresponding to crops showed in Fig 4.

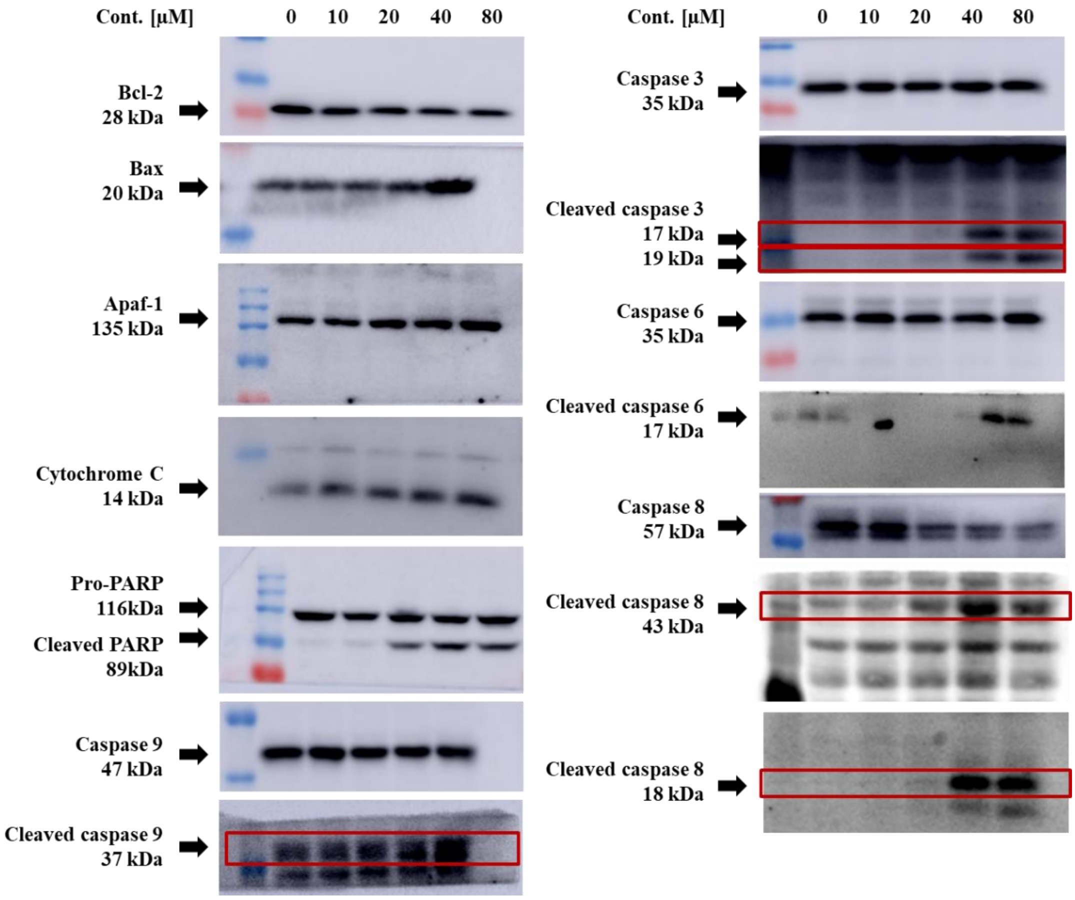

Fig. S8 Full-length blots corresponding to crops showed in Fig 5.

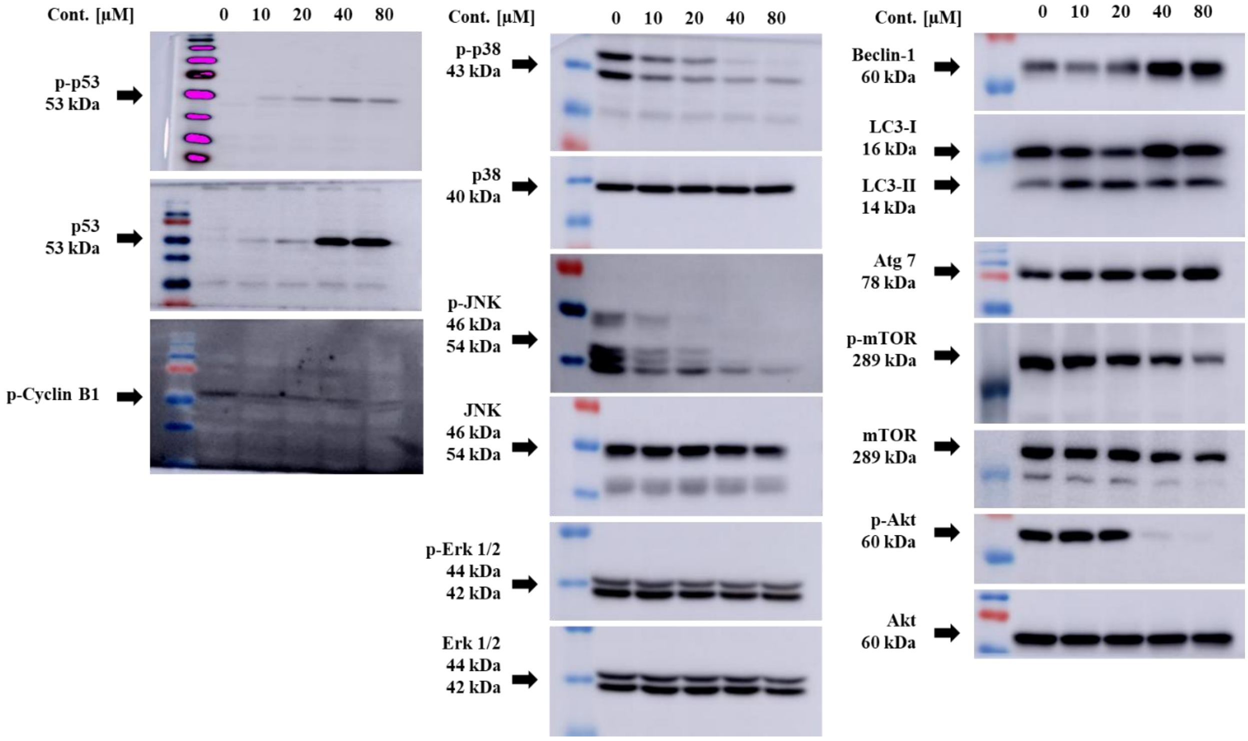

Supplement: Supplementary file 1 — Supplementary information [file 41598_2020_64871_MOESM1_ESM.pdf]
